# 

**Published:** 1891-10-31

**Authors:** 


**Notices of Births, Deaths, and Marriages**, are inserted in this column at a charge of 2s. 6d. for an announcement not exceeding four lines.

### NOTICE TO CORRESPONDENTS.

All MS., Letters, Books for Review, and other matters intended for the Editor should be addressed The Editor, The Lodge, Porchester Square, London, W.

The Editor cannot undertake to return rejected M.S., even when accompanied by stamped directed envelope.

**Notice to Advertisers and Subscribers.**

All Advertisements, Orders for Copies of The Hospital, and Business Communications should be addressed to The Publisher, *not to the Editor*, The Hospital, 140, Strand, W.C.

The Cost of Subscription, post free, is as follows:—Per week, 1½d.; per quarter, 1s. 8d.; per half-year, 3s. 3d.; per annum, 6s. 6d. Foreign:—Per annum, 8s. 8d.; payable, in all cases, in advance.

**"Burdett's Hospital Annual," 1891—1892.**

Third year of publication. 600 pp. Boards, gilt lettering. A most complete guide to British and Colonial Hospitals, Dispensaries, Nursing and Convalescent Institutions, and Asylums. Price 3s. 6d. Published by The Hospital (Limited), 140, Strand, W.C.

**NOTICE.—The Index for Vol. X. (ending September, 1891), is on sale at the Office of this Journal, price 2½d., post free.**

### GENERAL CHARITIES.

[This Column is devoted to an account of work of charitable institutions other than Medical Charities. The Editor will be glad to receive reports or news of work at 140, Strand. *Philanthropy* should be plainly written on the envelope.]

Among the smaller societies established for the benefit of the blind, the **HOME AND SCHOOL FOR BLIND CHILDREN**, Goldsmith's Place, Kilburn, does unostentatiously good work. The home is intended for quite young children, who are admitted between the ages of three and eight, boys being retained till the age of twelve. Many of the children are subsequently drafted into the Blind School, Upper Avenue Road. An annual sale is held every November, and the Lady Superintendent will be grateful if those who are interested in the work will attend and make purchases.

The claims of the **NATIONAL ORPHAN HOME** for support by the public were strongly advocated last week at a meeting held at the Mansion House, over which the Lord Mayor presided. The object of the Institution, which is situated on Ham Common, Richmond, is to receive orphan girls, who are maintained, educated, and prepared for domestic service. The Lord Mayor testified from personal knowledge to the excellent training which the children receive in the Home, to which any poor orphan is admitted, without consideration of creed or any restriction, provided it is shown that such orphan is poor and necessitous. At the present time there are about a hundred children on the books of the Institution, which has room for fifty more, but cannot accommodate this extra number for want of funds. The Committee desire additional annual subscriptions, rather than donations, so that they can have an income on which they can rely year by year. Those girls who can produce the best characters from the situations to which they have gone after leaving the Home receive prizes, which are provided by the interest obtained on a sum of £400 given for the purpose by Lady Peek. In this and other ways a link is maintained between the Home and its old inmates.

On the occasion of the recent visit of the Duchess of Albany to the **PRINTERS' ALMSHOUSES** at Wood Green, in order to declare the new extension of the buildings open, a strong tribute was paid both by Mr. Edward Lawson and Mr. W. A. Burdett-Coutts to the good work done in the Institution. The asylum now gives shelter to thirty-two aged and infirm printers and their widows, and each set of apartments is absolutely separate and independent. The management is fortunate in having practically defrayed already the cost of the new building towards which one thousand pounds have been given by an anonymous donor. A sum of £3,739 has also been contributed to this end from other sources, among them being four organizations of working printers called "auxiliaries." As Mr. Spottiswoode pointed out, what now remains is to provide an endowment fund for this excellent asylum. Such a fund has already been started on its way by contributions of £21 from the Company of Stationers; £20 (annual) from Mr. Henry Burt; £20 from Mrs. Vincent Figgins; £10 10s. from Mr. Alderman and Sheriff Tyler; £5 5s. from Mr. G. Singer; £210 from the South London Auxiliary; £200 from the Camden Town Auxiliary; and £100 from the City of London Auxiliary. By contributing to this fund, the public can best pay the debt it owes to printing, and to those who toil so laboriously in the art.

During the past year more men were received into the **NEWPORT MARKET REFUGE** than during the previous twelve months, whereas the number of women admitted to the Women's Refuge was less as compared with the men. In the case of the women the proportion of nights' lodging given was about a third more than that given to the men, owing to the fact that, out of the number passing through, one hundred and twenty were penitentiary cases. These have to be kept longer in the Refuge while the Sisters are finding suitable occupations for them or making the necessary arrangements for their future. The Committee appeal urgently to those who take an interest in penitentiary work to support them liberally in this branch of their work. It is hardly necessary to point out that the penitentiary branch of the Institution has much expense attached to it, if it is to be maintained in a satisfactory manner. One additional expense incurred in this particular department is caused by the necessity of providing the women, in most cases, with an outfit before the Sisters are able to place them in situations. The decision as to the eligibility of cases seeking admission to the Refuge is left in the hands of the Committee and Superintendent, and that they have not been wholly unsuccessful is perhaps best shown by the fact that many men have not remained in during the full seven days allowed, having found work after staying for two or three nights only in the Refuge. The method of selection adopted is as follows: Admission is never granted to those who seem only fit for the casual ward or infirmary owing to age or inability to work; pensioners are not admitted, excepting in cases of urgency; and every endeavour is made, as far as is possible, to exclude the loafing classes, who have no wish to work, and to select for admission *bonâ fide* working men who are temporarily out of employment. Though the Committee does not undertake to find work, every assistance and advice are given to the men in order to help them in obtaining it. In connection with the Institution is an excellent **INDUSTRIAL SCHOOL**, in which from eighty to one hundred boys receive a useful industrial training. The majority of the boys are placed in regimental bands.

### SCRAPS AND GLEANINGS.

A MUNICIPAL Fever Hospital is talked of for Leicester.

THE Leicester Hospital Saturday collection amounts to £2,124.

THE Hospitals Association is about to open twelve new ambulance stations.

DR. COGHILL, House Governor of the Birmingham General Hospital, has resigned his post.

A MOST successful concert was held last week at Enfield, in aid of the Cottage Hospital.

THE annual report of the Stratford-on-Avon shows a balance on the accounts in favour of the hospital.

THE Louth Infirmary has received £60, the result of the Hospital Saturday collection in that place.

HAWICK wants a fever hospital for the district, but it cannot quite make up its mind as to paying for it.

THIS is quite the day for Fever Hospitals. Ross-shire is now taking the subject up, and finds that it is most inadequately provided.

THE need of a Cottage Hospital is felt at Grays, Essex. A Church Parade in aid of this object was held, and a sum of £48 collected.

STEPS are being taken by a number of wealthy and benevolent persons in Philadelphia to build and endow a Consumption Hospital in that city.

THE St John's Hospital, Leicester Square, has just commenced a series of entertainments for the patients to be continued throughout the winter.

THE Hitchin Hospital Sunday Collection amounts to £18. This will go to the Hitchin Infirmary and the Hertfordshire Convalescent Home, St. Leonards.

THE friendly societies of Watford are endeavouring to supply a sufficient sum to the Cottage Hospital to warrant the abolition of patients' fees at the hospital.

AN interesting event took place on the 17th at Wood Green, on the occasion of the opening of the Printers' Almshouses by H.R.H. the Duchess of Albany.

A FELLOW of the Royal College of Physicians writes to the *Times* suggesting a pension for Dr. Collie, and points out the advantage of his remaining at the Eastern Hospital on the teaching staff.

THE "Old Students" annual dinner, in connection with the Charing Cross Hospital College of Medicine, took place at the Holborn Restaurant last week, under the presidency of Dr. A. W. Orwin.

PLEURO-PNEUMONIA has broken out at a dairy-farm in South London. Twenty years ago such an outbreak in the East of London caused the first importation of milk by rail from the country to London.

THE Kent and Canterbury Hospital is to be congratulated on its friends. Mrs. Coutts has contributed the sum of £1,000, "pour encourager les autres" with such effect that the deficit fund now stands at £2,175.

MRS. CARR-BENTON has given £550 to the Brighton, Hove, and Preston Dispensary on condition that two houses about to be purchased should be devoted to extending the Carr-Benton Wing of a branch dispensary in Sackville Road.

THE Duke of Edinburgh paid a visit to Bristol on the 23rd inst., where he opened the new wing of the General Hospital, and took part in a function at the Knowles Industrial Home, and led the strings at a concert in aid of local charities.

AN additional storey has been added to a wing of the Stamford Infirmary. This provides much needed accommodation for nurses who formerly slept in the attics. Two extra ward maids have been engaged to lighten the nurses' labour.

AT the adjourned meeting of the governors of Addenbrooke's Hospital the report of the polling on the amendment proposed, with reference to the surgical staff, was read. The amendment was lost, and the original proposition was carried *nem. con.*

THE question of hospital accommodation has arisen again at Huddersfield Four years ago a site was bought for a hospital; nothing but a war of words has raged ever since. One member of the Town Council means business; let us hope he will gain the day.

WHAT may be called the Requiem of the Derbyshire Royal Infirmary as it now stands took place on the 23rd inst. A special service and meeting was held. This was the eighty-second anniversary of the institution. £49,000 had been collected towards the new building.

THE proposed extension of the Edinburgh Royal Infirmary is under serious consideration. The addition is estimated at £73,000. The Treasurer does not think the managers need hesitate from financial reasons, considering the popularity of the institution.

THE Beverley Guardians have not done quarrelling over the Infirmary question; but they have at last come to two decisions. Firstly, not to spend more than £2,000; and secondly, to get an architect's estimate. This is the result of over two years' consideration and debate.

GREAT indignation is felt by the treatment of the dead body of a patient who died in the Poplar Hospital from the results of an accident. All clothing was removed from his body, and he was placed nude in the public mortuary. It appears that the Poplar Hospital has no room available for inquests.

THE explanation given by the Poplar Hospital authorities of the disgraceful occurrence we have mentioned is that the undertaker who had charge of the burial clothes of the deceased was superseded unexpectedly by the undertaker from the Coroner's office, who was allowed to remove the body, by the assistant porter, with whom the fault lies.

THE Holborn Guardians find there has been of late an extraordinary increase in the quantity of wines, spirits, and tea consumed in the Mitcham Workhouse. Brandy has risen six fold in the weekly bill, and wine from 220 to 670 ounces. It appears that the explanation rests with the workhouse medical attendants.

THE new infectious diseases hospital erected by the Pudsey, Calverley, Farsley, Idle, and Eccleshill Local Boards jointly, is now on the point of completion. The buildings have been erected from designs prepared by Messrs. W. and J. B. Bailey, architects, Bradford. The site is just outside the borough of Bradford, and is easily accessible from all the five local board districts which it is intended to serve.

### The Student of Medicine.

[All communications for this Supplement should be addressed to The Editor of The Hospital, at 140, Strand, with the words, "Students' Column," written in the left hand top corner of the envelope.]

### APPOINTMENTS.

At the Medical School of Charing Cross Hospital, Archie J. Collum, L.R.C.P., M.R.C.S., has been appointed junior demonstrator of anatomy. At the South Infirmary and County Hospital, Cork, C. A. Harvey, M.D., M.Ch.Dub., has been appointed extern physician. At the Cheshire County Asylum, Stuart Gerald McAllum, M.B., C.M., Edin., has been appointed junior assistant medical officer. At the City of London Hospital for Diseases of the Chest, Victoria Park, E., Charles H. McIlraith, M.A., M.B., C.M., Glasgow, has been appointed house physician. At the Rotherham Hospital T. G. McKellar, M.B., C.M., has been appointed house surgeon. At the Belgrave Hospital for Children C. R. Martin, L.R.C.S., has been appointed house surgeon. At the Royal Free Hospital, Gray's Inn Road, C. S. Pethick, M.A.Camb., M.R.C.S., L.R.C.P., has been appointed junior resident medical officer. At the Walsall Cottage Hospital Kenneth W. Poole, M.B., B.S. Durham, M.R.C.S., L.R.C.P., has been appointed resident house surgeon. At the Belfast Union Hospital, A. Gardner Robb, M.B., B.Ch., has been appointed house surgeon. At the Royal National Hospital for Consumption, Ventnor, Herbert M. Wood, M.R.C.S., L.R.C.P., has been appointed resident medical officer.

### VACANCIES.

The following vacancies are announced this week, the amount of salary in each case being given after the name of the institution:

### Resident Medical Officers.

Berks Asylum, Moulsford, Second Assistant Medical Officer—Salary £100, with board, &c.
Cancer Hospital (Free), S.W., House Surgeon—Salary £50, with board, &c.
East London Hospital for Children, Shadwell, E., House Physician—Board, &c.
London Fever Hospital, Assistant Resident Medical Officer.
National Hospital for Paralysed and Epileptic, Queen Square, House Physician—Salary £60, and board, &c.
North-West London Hospital, Resident Medical Officer and Assistant Resident Medical Officer—Salary for the senior, £50 for six months.
Paddington Infirmary, Resident Clinical Assistant—Board, &c., honorarium of 12 guineas for six months.
Ripon Dispensary and Cottage Hospital, Resident House Surgeon and Dispenser—Salary £70, and board, &c.
Royal Alexander Hospital for Sick Children, Brighton, House Surgeon—Salary £80, with board, &c.
Seamen's Hospital Society, Greenwich, House Surgeon—Salary £50, with board, &c.
Sheffield Public Hospital and Dispensary, Assistant House Surgeon—Salary £50, with board, &c.; also House Surgeon—Salary £50, and board, &c.

### Non-Resident Medical Officers.

Dental Hospital of London, Leicester Square, Dental Surgeon and Assistant Dental Surgeon.
Guy's Hospital, Assistant Anæsthetist.
Hospital for Sick Children, Great Ormond Street, Medical Registrar and Pathologist—Honorarium 50 guineas.
Royal Hospital for Diseases of the Chest, City Road, Physician.

### NOTES ON INSTRUMENTS.

Advanced Students' Microscope.—We have had opportunities of examining a microscope made by W. Johnson and Sons, of 188, Tottenham Court Road, and which is called the Advanced Students' Microscope. This machine is constructed especially for bacteriological and high power work, and is provided with a new substage adjustment. The general shape and size of the stand is after the pattern adopted by Zeiss, and followed by nearly all the modern makers, which is undoubtedly the best and most convenient form. The whole being very compact and easily carried about from place to place; the smaller machines of the present day having quite replaced the large and cumbersome older kinds, which were often very attractive to the amateur worker on account of the imposing appearance of the instrument. The chief characteristic of the machine we are considering is the adoption of a screw movement to adjust the substage, in place of the usual rack and pinion. Thus there is a very ready and easily workable adjusting apparatus, carrying the Abbé condenser and other apparatus. By this arrangement the condenser can be brought up or down or replaced by a simple or convex mirror, without disarranging the stage of the microscope and any object that may be under observation. This piece of mechanism is a distinct advantage over the German instruments, which have to be disturbed if the condenser is required to be removed. If, when examining an object with one of the usual pattern of modern microscopes, with the Abbe condenser attached, it is desired to examine the object without the condenser the whole stage of the machine has to be tilted up and the object removed, to be again replaced when the simple mirror has been fixed. It will thus be seen the machine introduced by Mr. Johnson is a distinct advance on the older methods. Some other points about the microscope are, the substage with its fittings is fixed to the microscope, and is free from lateral movement; by a simple arrangement it can be removed or replaced. The mirror can also be removed for direct light. The substage, with the patent screw adjustment and mechanical centreing arrangement carrying an Abbe condenser, is mounted on a substantial tail-piece and slides in dovetailed fittings. The body is constructed with lengthening tube with graduations marked for Continental or English objectives. The coarse adjustment is the improved oblique rack and pinion principle, which ensures equality of motion, working in truly planed dovetailed fittings and having full-sized milled heads. The fine adjustment is the most approved form, giving great delicacy of adjustment, with freedom from lateral movement, moving the whole of the body, and thus maintaining a uniform distance between the ocular and objective. The mirrors are plain and concave, mounted on a swinging arm, sliding in a dovetailed groove, and they can readily be removed for direct light.

### STUDENTS' MEDICAL SOCIETIES.

### Westminster Hospital.

The monthly meeting of the Guthrie Society was held on Thursday, October 8th, at eight p.m. Dr. Allchin presided, supported by Messrs. Yearsley, Mills, Campbell, Morris, Winckworth, and the Secretary. The following were elected members of the Society: Messrs. Walker, Towne, C. Farrant, Watcher, Cropp, Gordon Green. Then followed a very successful smoking concert, which had been arranged by Messrs. Mills, Campbell, and the secretary. Considerable talent was exhibited during the evening. Advantage was taken by the gathering to bid a good-bye to Dr. E. W. Witham, who is leaving for Calcutta. The President's words of farewell were most touching, and the pleasant evening concluded by the rendering of "Auld Lang Syne."

### University College Medical Society.

The annual public night of the above Society was held on Wednesday, October 7th. The chair was taken in the Botanical Theatre of the College by the President, Mr. G. B. M. White, B.S., F.R.C.S. The address was delivered by Dr. Gaskell, F.R.S., the subject being "On a New Theory of the Origin of Vertebrates Derived from the Study of Vertebrate Anatomy and Physiology." Dr. Gaskell propounded at some length the theory which is now known to most biologists, with many new facts, as yet unpublished, and all tending to demonstrate the evolution of the lower vertebrates from a crustacean ancester. There was a large and appreciative audience composed of students and many eminent scientific men. The Flaxman Gallery and Anatomy Museum was thrown open, and there was a demonstration of microscopic specimens in the Mathematical Theatre. Messrs. Martindale and Co., exhibited some recent therapeutic agents of special interest.

### SCHOLARSHIPS AND PRIZES.

### Entrance Scholarship—October, 1891.

University College Hospital: *Entrance Exhibitions*—W. B. L. Trotter, £100; C. Goring, £60; A. J. Rodocanachi, £40; and J. C. H. Leicester, £40.

Westminster Hospital Medical School.—The Entrance Scholarship in Arts, value £80, has been awarded to F. Riley.

The London Hospital Medical College.—The following are the names of the successful competitors for our *Entrance Scholarships*:—First Entrance Science Scholarship, value £75, Herbert Innes; Second Entrance Science Scholarship, value £50, E. C. Davenport; First Buxton Scholarship, value £30, G. H. Warren; Second Buxton Scholarship, value £20, J. G. Wallis.

St. Mary's Hospital.—The following candidates have been successful in obtaining *Entrance Scholarships* at the above school: Natural Science Scholarships—B. Lewitt, W. B. Stevenson, and H. Sugden; University Scholarships—J. A. Wright, B.A., and W. J. Harris, B A.

Charing Cross Hospital Medical School.—The Entrance Scholarship, value 100 guineas, has been awarded to J. R. Langley, and that of 50 guineas to Howard Green.

St. Mary's Hospital, Paddington.—Stanley B. Atkinson, second son of the Rev. J. W. Atkinson, of Latimer Congregational Church, Stepney, has just secured in open competition the fifty guinea scholarship awarded at this hospital.

### PASS LISTS.

### Examining Board in England by the Royal Colleges of Physicians and Surgeons.

The following gentlemen passed the Second Examination of the Board in the subjects indicated at a meeting of the Examiners on Monday, the 5th inst.:—

Anatomy and Physiology.—H. Alston, of Sydney University; C. H. Caldicott, A. E. Tonks, R. F. Ryland, H. B. Munshull, M. L. G. Hallwright, and W. Chapman, of Queen's College, Birmingham; W. E. Evans, of Dublin; W. T. Lydall, of Bristol Medical School; R. Crossley, of Owens College, Manchester; W. J. Boyes and W. J. Gregerson, of Melbourne University; W. A. Michie, of Aberdeen University and Guy's Hospital; H. M. Williamson, of McGill College, Canada; L. A. Smith, of London Hospital.

Anatomy only.—D. Maguire, of Queen's College, Galway; W. Mansergh, of Owens College, Manchester; H. C. W. Wood, of Queen's College, Birmingham; R. G. Worger, of Bristol Medical School and Mr. Cooke's School of Anatomy and Physiology; J. A. W. Pereria, of Grant Medical College, Bombay.

### THE STUDENT OF MEDICINE—(*continued*).

Physiology.—W. J. Bowden, H. H. Oram, and G. G. Joynson, of Owens College, Manchester; F. Husband and J. H. R. Pigeon, of Bristol Medical School; W. Mettam, of Sheffield Medical School; W. V. P. Teague, of London Hospital.

Passed on the 6th inst.:—

Anatomy and Physiology.—B. L. Abraham. of University College.

Anatomy only.—T. B. Abbott, of Yorkshire College, Leeds; L. Bostock, of Owens College, Manchester; W. C. Pitt and W. E. Pain, of Guy's Hospital; R. Slocock, C. F. Poole and A. McD. Cowie, of St. Thomas's Hospital and Mr. Cooke's School of Anatomy and Physiology; C. C. Williams, J. E. S. Old, and G. Lewis, of London Hospital; F. P. Rose, of London Hospital and Mr. Cooke's School of Anatomy and Physiology; C. W. Lamphier, of Charing Cross Hospital; H. W. Armit and R. W. Gilmour, of St. Bartholomew's Hospital;

Physiology Only.—S. C. Legge G. R. Northwood, and L. Lowe, of Queen's College, Birmingham; M. C. Barber and A. T. Morgan, of Bristol Medical School; G. A. Child, of Oxford University; I. Watts, of Owens College, Manchester; F. W. Bailey, of University College, Liverpool; H. W. Carson and A. C. B. Casson, of St. Bartholomew's Hospital; A. Emlyn and W. Drake, of University College; E. Moore, of St. Thomas's Hospital and Mr. Cooke's School of Anatomy and Physiology; L. J. Miskin, of St. Thomas's Hospital; H. F. Humphreys, of Guy's Hospital; E. R. Badcock and R. G. Jones, of London Hospital; W. H. Reed, of King's College; P. L. Watkins-Williams, of Middlesex Hospital and Mr. Cooke's School of Anatomy and Physiology.

### University of Durham: Faculty of Medicine.

The following candidates have received degrees, &c., as indicated:—

Degree of Doctor in Medicine for Practitioners of Fifteen Years Standing.—G. Bainbridge, M.R.C.P.Lond.; E. A. Fox, L.R.C.P., L.R.C.S. Edin.; S. H. Hobley, L.R.C.P. Lond., M.R.C.S., L.S.A.; H. A. Lawton, M.R.C.S., L.R.C.P., D.P.H. Lond.; J. A. Masters, M.R.C.P. Lond.; B. S. Ringer, M.R.C.S., L.S.A.; A. P. Sherwood, M.R.C.S., L.R.C.P. Lond.; H. Smalley, M.R.C.S., L.R.C.P. Lond.; T. Walby, M.R.C.S., L.R.C.P. Edin.

Degree of Doctor in Medicine.—F. Bass, M.B.Durh., L.R.C.P. Lond., F.R.C.S Eng.; C. Gayford, M.B.Durh., M.R.C.S., L.R.C.P. Lond.; R. Heelis, M.B.Durh., M.R.C.S., L.R.C.P.Lond.; L. W. Keiffenheim, M.B., B.S.Durh., M.R.C.S., L.R.C.P.Lond.; R. G. P. Lansdowne, M.B., B.S.Durh.; F. W. Ramsay, M.B., B.S.Durh; F. P. Wightwick, M.B.Durh., M.R.C.S., L.R.C.P., D.P.H.Lond.

Degree of Bachelor in Medicine.—*Honours* (*Second Class*): F. W. Gunn, M.R.C.S., L.R.C.P., L.S.A., King's College Hospital; T. Carr, M.R.C.S., L.S.A., Guy's Hospital; T. L. Bryan, College of Medicine, Newcastle-on-Tyne. *Pass List*: T. M. Allison, R. H. Cole, C. V. Dingle, W. J. Durant, T. G. Hall, F. Hunton, J. P. Iredale, J. A. Kendall, J. Law, and A. E. Neale, all of the College of Medicine, Newcastle-on-Tyne; E. W. P. Baines, St. George's Hospital; J. V. Blachford, Guy's Hospital; Appu-Hennedige C. De Silva, M.R.C.S., L.R.C.P., Ceylon Medical College; R. B. Duncan and A. A. Fennings, both of St. Mary's Hospital; H. D. Johns, M.R.C.S., L.R.C.P. Lon., Charing Cross Hospital; C. Meadon, British Medical School; E. Rye, Owens College, Manchester; W. R. Thurnam and A. M. Wilson, both of St. Thomas's Hospital.

Degree of Bachelor in Surgery.—T. M. Allison, C. V. Dingle, W. J. Durant, T. G. Hall, F. Hunton, J. P. Iredale, J. A. Kendall, J. Law, and A. E. Neale, all of the College of Medicine, Newcastle-on-Tyne; E. W. P. Baines, St. George's Hospital; J. V. Blachford, Guy's Hospital; Appu-Hennedige C. De Silva, M.R.C.S., L.R.C.P., Ceylon Medical College; R. B. Duncan and A. A. Fennings, both of St. Mary's Hospital; F. W. Gunn, M.R.C.S., L.R.C.P., L.S.A., King's College Hospital; H. D. Johns, M.R.C.S., L.R.C.P.Lond., Charing Cross Hospital; W. R. Thurnam and A. M. Wilson, both of St. Thomas's Hospital.

Degree of Bachelor in Hygiene.—T. Buckham, M.B., B.S. Durh.; W. H. Turnbull, M.B., B.S. Durh.

Licence in Sanitary Science.—F. W. Gunn, M.R.C.S., L.R.C.P., L.S.A.

### Society of Apothecaries' of London.

The following candidates have passed the recent examinations in the subjects indicated:—

Surgery.—J. E. Bailey, Univ. Coll. Hosp.; P. C. Bardsley, Cambridge and Univ. Coll. Hosp.; W. T. Davies, London Hosp.; H. W. Roberts, St. George's; C. M. Wheeler, Royal Free.

Medicine, Forensic Medicine, and Midwifery.—C. W. Allen, St. Mary's; H. S. Cooper, St. George's; P. C. De Wet and W. S. McGeagh, St. Thomas's; C. M. Wheeler, Royal Free.

Medicine and Forensic Medicine.—J. S. Newington, Edin. Univ.; W. R. Nicol, Montreal.

Medicine and Midwifery.—H. B. T. Symons, Aberdeen Univ. and Charing Cross Hosp.

Forensic Medicine.—V. H. Barr. Guy's; A. C. Fenn, St. Bartholomew's.

To Messrs. Cooper, De Wet, McGeagh. Newington, Nicol, Roberts, and Symons, and to Miss Wheeler, was granted the Diploma of the Society, entitling them to practise medicine, surgery, and midwifery.

At the examination in Arts, the following passed in all the subjects required for registration: First Class (in order of merit).—G. B. Brown, A. R. Heath, S. W. St. Cedd, J. N. Walker.

### EVENTS FOR THE MONTH OF OCTOBER.

#### Football.

St. Mary's Hospital.—October 31st, v. Charlton, at Woolwich; second fifteen, October 31st, v. Eltham House, at home.

St. Thomas's Hospital.—First fifteen, October 31st, v. Swansea, at Swansea. Second fifteen, October 31st, v. Clapham Rovers II., at Lambeth.

### The Hospital Advertiser.

**THE HOSPITAL FOR SICK CHILDREN,**

